# Supplementary material for: Dynamic association of H3K36me3 with pericentromeric heterochromatin regulates its replication time
Source: EMBO Rep. 2025 Sep 8;26(20):4950–76. doi: 10.1038/s44319-025-00575-6 (PMC12549960; doi:10.1038/s44319-025-00575-6)
Supplement: Supplementary file 10 — Expanded View Figures [file 44319_2025_575_MOESM10_ESM.pdf]

Expanded View Figures

Figure EV1. Cell cycle-dependent dynamics of the histone modification levels.

(**A**) Asynchronously growing cell populations pulsed with EdU are stained for EdU and histone modifications. After high-throughput imaging, the images were imported to FiJi, and the StarDist plugin was used to segment nuclei, followed by intensity measurement of DNA content, EdU, and histone modifications. The cell populations are sorted into (non)replicating using the mean EdU intensity; the non-replicating cells are divided into G1 and G2 based on the DNA content from the DAPI sum intensity. The example boxplot shows the corresponding histone modifications sum intensities normalized to DNA content and further normalized to G1 to see the fold change. The p-values inferred from ANOVA followed by Tukey's honest significant difference test are: G1:S = 0.004971475, S: G2 = 3.593188e-33, G1:G2 = 3.149946e-12. $n = 4730$ (three biological replicates). The boxplot shows the median (line), interquartile range (box: 25th to 75th percentile), and whiskers extending to data within 1.5× the IQR. n.s. not significant; *$p < 0.05$; **$p < 0.005$; ***$p < 0.0005$. (**B**) The plot shows the fold changes (normalized to G1) in the normalized histone modification intensity across different cell cycle phases for various histone modifications in mouse embryonic stem cells (mESCs). Statistical significance was assessed using a pairwise *t*-test (all *p* values are provided in the source data files). Asterisks indicate significant differences in normalized histone modification intensity between the specified cell cycle phases: n.s. no significant, *$p < 0.05$, **$p < 0.05$, and ***p < 0.005. In this figure, statistical significance is represented as * for any pairwise comparison where the difference is significant, and n.s. for not significant. Three independent biological replicates were pulled together after cell cycle classification for each histone modification. $n = 59{,}934$ from all histone modifications. (**C**) A table summarizes the significantly changed histone modification levels through the cell cycle. The amounts of the transcription, enhancer, and nascent chromatin-associated histone modification marks are increased in the S phase, whereas heterochromatin marks are decreased. Not all the histone modifications are recovered post-replication. Source data are available online for this figure.

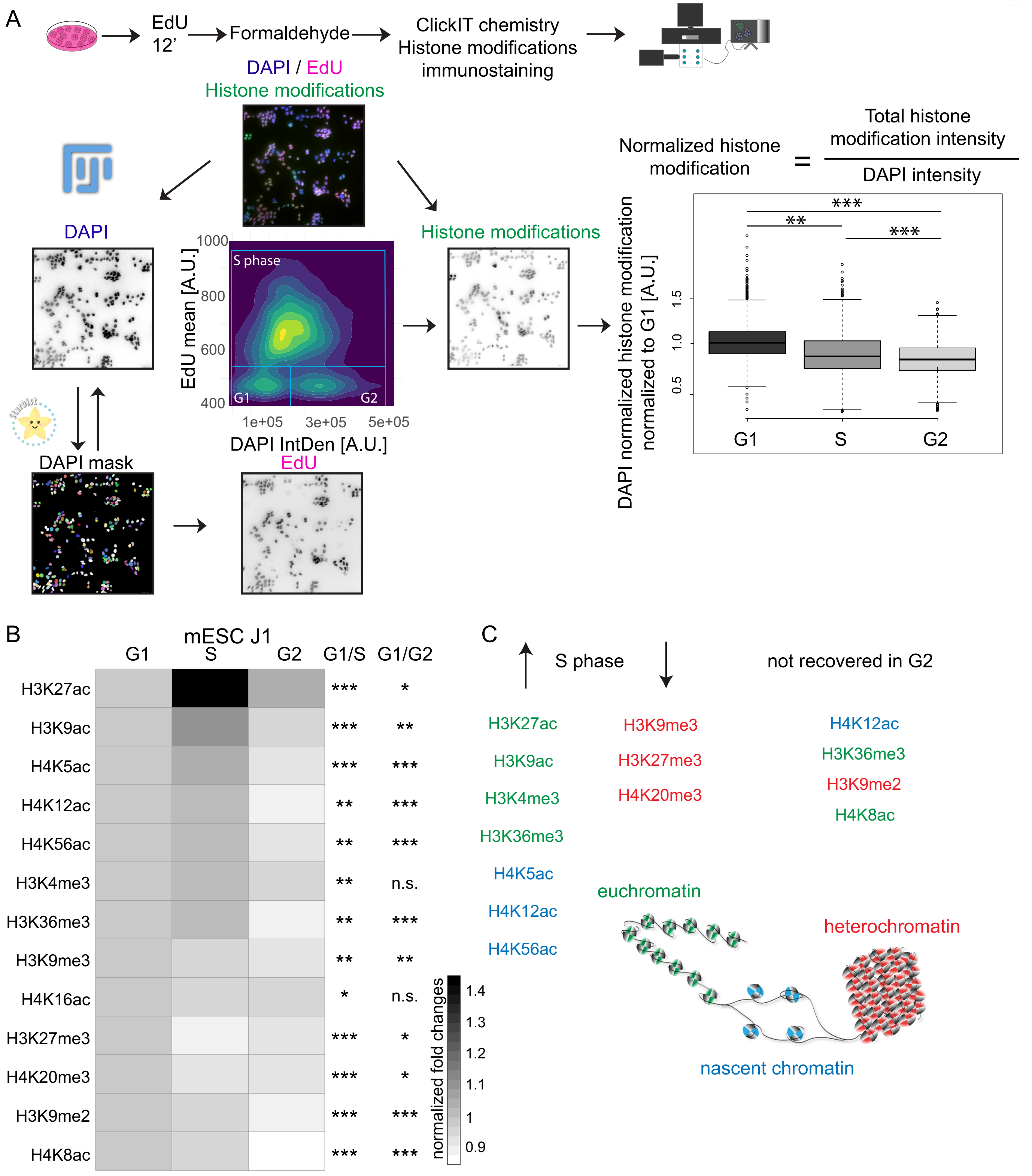
A
EdU 12'
Formaldehyde
ClickIT chemistry
Histone modifications
immunostaining
DAPI / EdU
Histone modifications
DAPI
S phase
G1
G2
EdU mean [A.U.]
1000
800
600
400
1e+05
3e+05
5e+05
DAPI IntDen [A.U.]
Histone modifications
DAPI mask
EdU
Normalized histone modification
=
Total histone modification intensity
DAPI intensity
DAPI normalized histone modification normalized to G1 [A.U.]
0.5
1.0
1.5
***
**
***
G1
S
G2
B
mESC J1
G1
S
G2
G1/S
G1/G2
H3K27ac *** *
H3K9ac *** **
H4K5ac *** ***
H4K12ac ** ***
H4K56ac ** ***
H3K4me3 ** n.s.
H3K36me3 ** ***
H3K9me3 ** **
H4K16ac * n.s.
H3K27me3 *** *
H4K20me3 *** *
H3K9me2 *** ***
H4K8ac *** ***
normalized fold changes
1.4
1.3
1.2
1.1
1
0.9
C
S phase
not recovered in G2
H3K27ac
H3K9ac
H3K4me3
H3K36me3
H4K5ac
H4K12ac
H4K56ac
H3K9me3
H3K27me3
H4K20me3
H4K12ac
H3K36me3
H3K9me2
H4K8ac
euchromatin
heterochromatin
nascent chromatin

 © The Author(s)

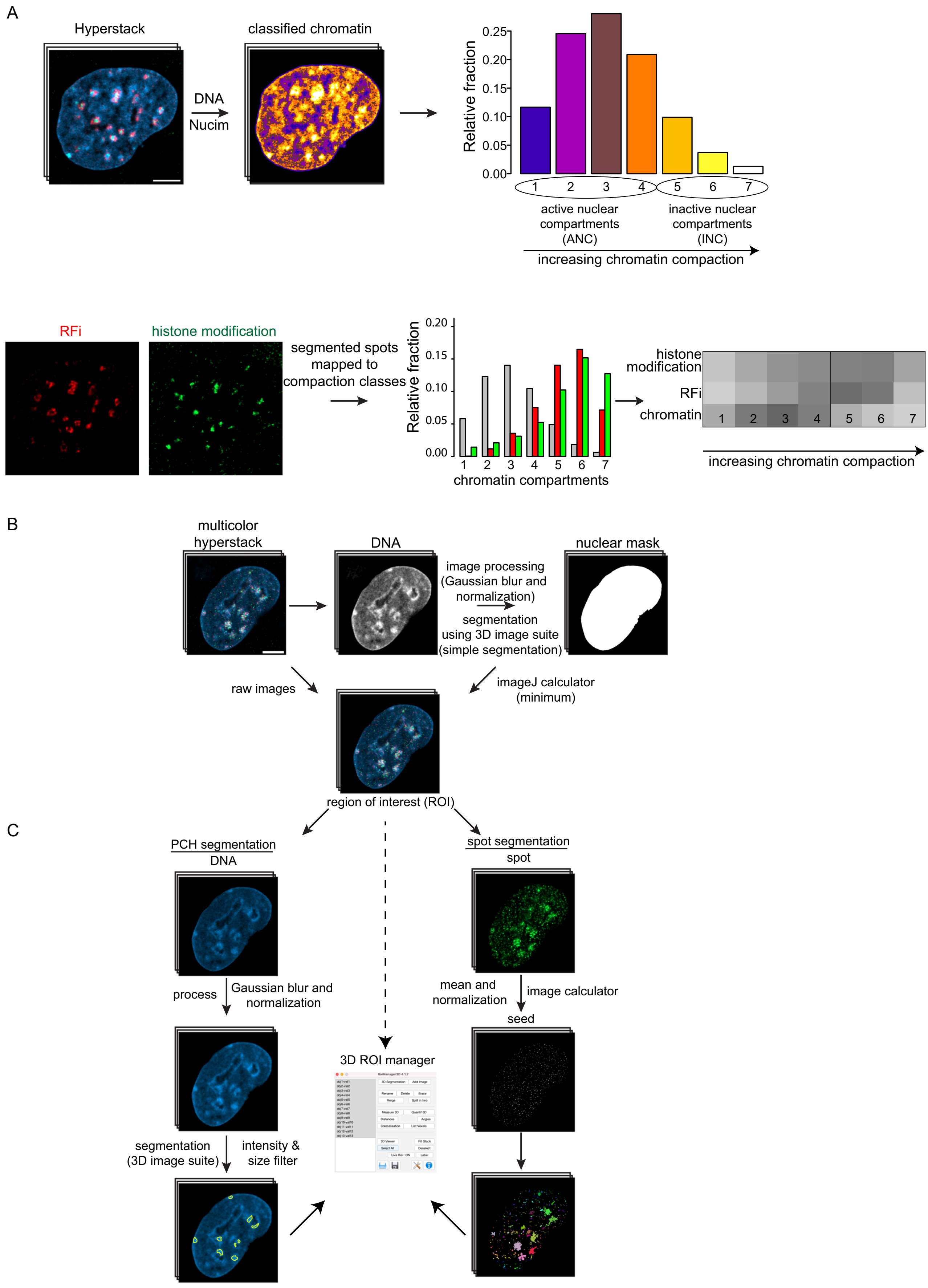
A
Hyperstack
DNA
Nucim
classified chromatin
Relative fraction
0.25
0.20
0.15
0.10
0.05
0.00
1 2 3 4 5 6 7
active nuclear compartments (ANC)
inactive nuclear compartments (INC)
increasing chromatin compaction
RFi
histone modification
segmented spots mapped to compaction classes
Relative fraction
0.20
0.15
0.10
0.05
0.00
1 2 3 4 5 6 7
chromatin compartments
histone modification
RFi
chromatin
1 2 3 4 5 6 7
increasing chromatin compaction
B
multicolor hyperstack
DNA
image processing (Gaussian blur and normalization)
segmentation using 3D image suite (simple segmentation)
nuclear mask
raw images
imageJ calculator (minimum)
region of interest (ROI)
C
PCH segmentation
DNA
process
Gaussian blur and normalization
segmentation (3D image suite)
intensity & size filter
3D ROI manager
spot segmentation
spot
mean and normalization
image calculator
seed

◀ **Figure EV2. High-resolution image analysis pipelines.**

(**A**) The pipeline shows the approach for chromatin compaction classification and mapping RFi/histone modification to different compaction classes using the statistical image analysis tool Nucim on the R platform. Individual voxels inside the nuclei are assigned to different compaction classes (increasing compaction from 1 to 7), where the first four represent active nuclear compartments, and the last three classes represent inactive nuclear compartments. The relative fraction of RFi/histone modifications in each chromatin class is measured. (**B**) The pipeline shows the approach to segmenting the nuclei and defining the region of the nuclei. The DAPI channel (DNA) was first processed with a Gaussian blur and was normalized using the stack histogram. Using the 3D ImageJ suite plugin, the processed image was segmented (simple segmentation, with size filter). The size selection removes small debris that might be present outside the nuclei. The 3D mask was binarized, and the image calculator (minimum) operation was used between the DNA mask and the channel of interest (including original DNA) to remove the signals outside the nuclei. (**C**) The scheme shows approaches to segment the pericentromeric heterochromatin (PCH) or spots (e.g., histone modifications/RFi). The PCH was segmented using a high intensity and volume threshold filtering in only the DAPI-dense regions. For RFi/RNA Pol II/histone modifications, a combined approach of 3D local maxima and intensity was used. First, the 3D local maxima of the images were extracted and used as seeds, around which an intensity threshold was applied to segment the 3D spots. For spot segmentation, the 3D ImageJ suite was used (spot segmentation, local threshold method = Gaussian fit, watershed = yes). All the masks were imported into the 3D ROI manager for further quantification/colocalization measurements. Scale bar: 5 µm.

© The Author(s)

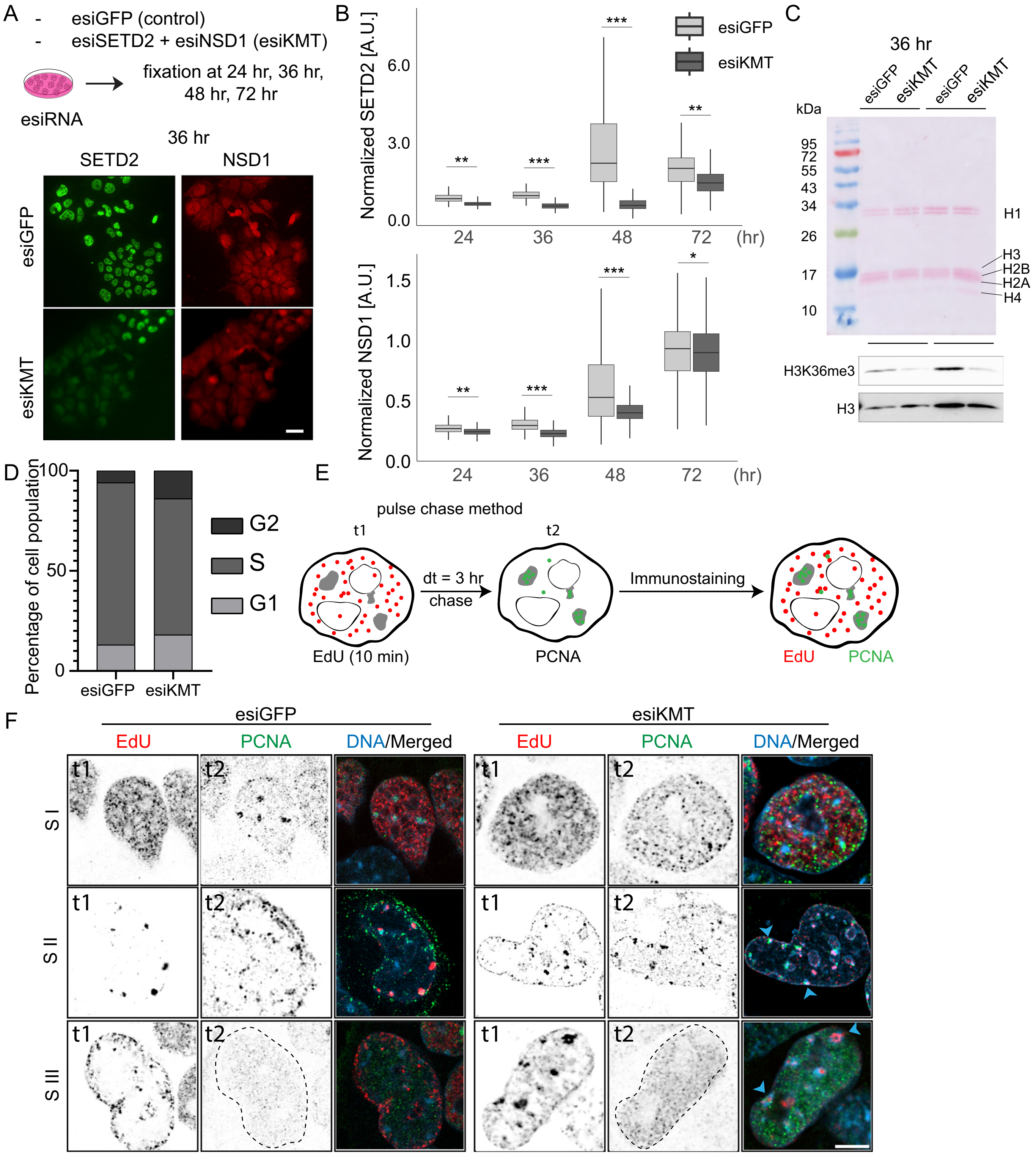
A
- esiGFP (control)
- esiSETD2 + esiNSD1 (esiKMT)
esiRNA
fixation at 24 hr, 36 hr, 48 hr, 72 hr
36 hr
SETD2
NSD1
esiGFP
esiKMT
B
Normalized SETD2 [A.U.]
Normalized NSD1 [A.U.]
esiGFP
esiKMT
24
36
48
72
(hr)
C
36 hr
esiGFP
esiKMT
kDa
H1
H3
H2B
H2A
H4
H3K36me3
D
Percentage of cell population
G2
S
G1
E
pulse chase method
t1
t2
dt = 3 hr chase
Immunostaining
EdU (10 min)
PCNA
EdU
F
esiGFP
esiKMT
EdU
PCNA
DNA/Merged
S I
S II
S III

Figure EV3. Knockdown strategy to reduce H3K36me3 using RNA interference, and its effect on replication timing.

(**A**) The knockdown of two K36-specific methyltransferases (KMT), SETD2 and NSD1, was performed using esiRNA, and the level was quantified at different time points using high-throughput imaging and analysis. Example images show the reduced levels of SETD2 and NSD1 in the nucleus. Scale bar:10 µm. (**B**) A significant reduction of SETD2 and NSD1 levels was observed from 36 to 48 h, while the effect was less prominent around 72 h. *n* (eGFP) = 6021, *n* (SETD2 + NSD1) = 6212. Cell images were used from three biological replicates. Relevant *p* values are inferred from pairwise Wilcoxon test between esiGFP and esiKMT: SETD2 (24 h: 0.00091, 36 h: 0.00021, 48 h: 0.00019, 72 h: 0.00058), NSD1 (24 h:0.00073, 36 h: 0.00042, 48 h: 0.00032, 72 h: 0.00059). The boxplot shows the median (line), interquartile range (box: 25th to 75th percentile), and whiskers extending to data within 1.5× the IQR. Statistical significance was performed using the Wilcoxon test (not significant (n.s.) is given for *p* values ≥0.05; one star (*) for *p* values <0.05 and ≥0.005; two stars (**) is given for values <0.005 and ≥0.0005, and 0.0005 to 0 are given (***). (**C**) The knockdown of H3K36me3 was validated using acid-extracted histones between GFP and KMT knockdown samples. The ponceau (upper blot) shows the acid-extracted histone. On the lower panels blot shows the detected histone H3 along with H3K36me3. (**D**) The plot shows the percentage of the S-phase population among esiGFP and esiKMT measured from high-throughput image analysis using EdU and DNA content. Upon knockdown of KMTs, a relatively lower population was detected in the S phase. *n* (esiGFP) = 1202, *n* (esiKMT) = 1461, merged from two biological replicates. (**E**) The illustration shows the pulse-chase method to capture the order of RFi patterns to infer replication timing. S phase spatial patterns S I–S III were inferred based on EdU incorporation and PCNA spatial patterns: S I (EdU-negative, PCNA-positive), S II (early EdU labeling with characteristic PCNA distribution), and S III (EdU-positive with loss of punctate PCNA foci). (**F**) Images show the results of a pulse-chase experiment to capture the spatiotemporal dynamics of genome replication. In the control, the replication progresses from euchromatin to constitutive heterochromatin, followed by lamin-associated domains. In KMT, knockdown, the pericentromeric heterochromatin (marked by blue arrow), and lamin-associated domains are replicated concomitantly after euchromatin is replicated, *n* (esiGFP) = 12, *n* (esiKMT) = 14. Scale bar: 5 µm. Source data are available online for this figure.

 © The Author(s)

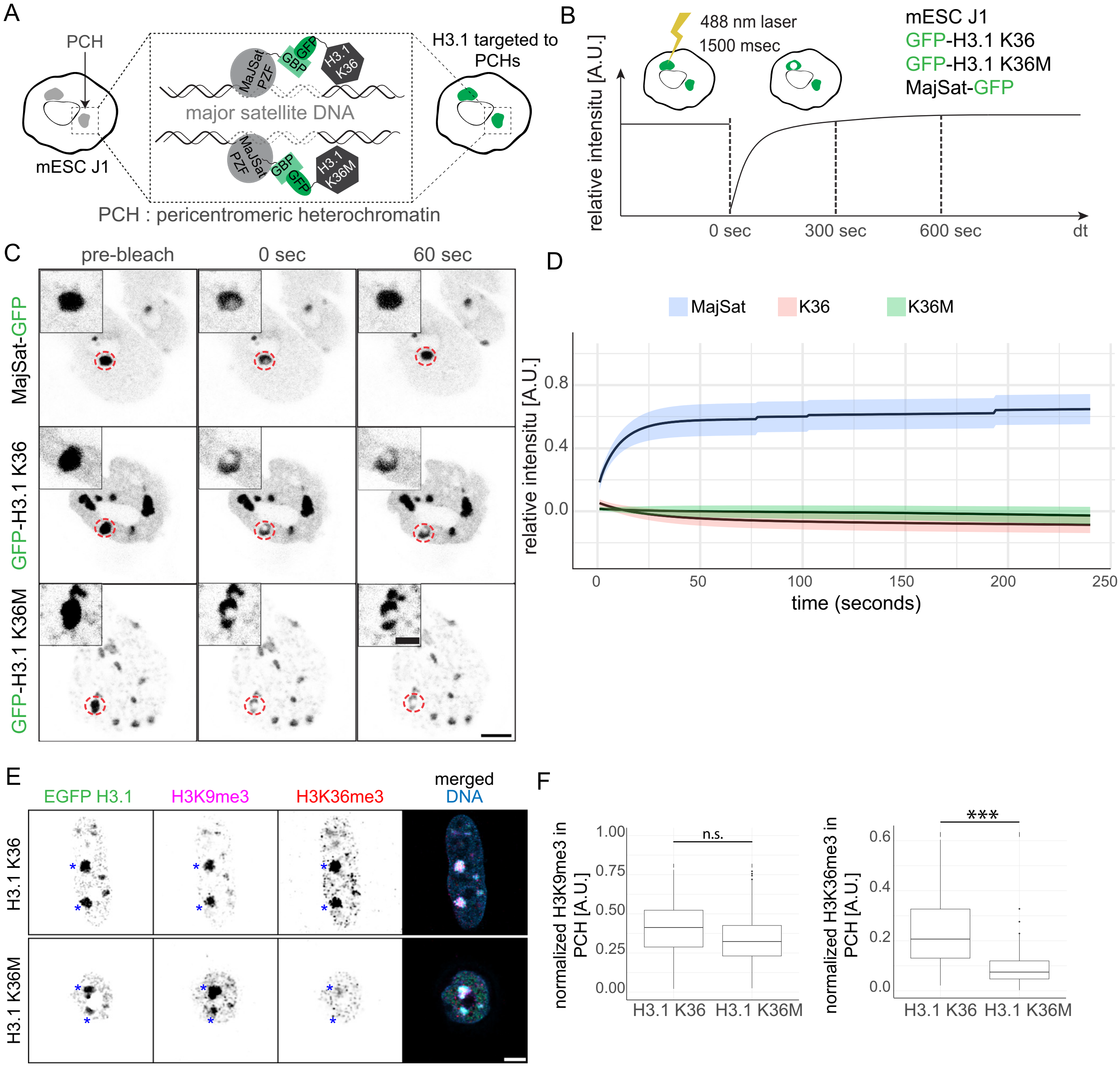

Figure EV4. Knockdown strategy to reduce H3K36me3 from pericentromeric heterochromatin (PCH) using oncohistone H3.1 K36M.

(**A**) The illustration shows the targeting strategy of H3.1 or H3.1 K36M tagged with GFP to the PCH using PZF-MajSat nanobody fusion protein to reduce the level of H3K36me3 locally from the PCH by taking advantage of the replication-dependent H3.1 incorporation. (**B**) The illustration shows the fluorescence recovery after photobleaching (FRAP) strategy to gauge the dynamics of H3.1 after incorporation. (**C**) Images show the pre-bleach and post-bleach dynamics of H3.1 (H3.1 K36 and H3.1 K36M), with MajSat-GFP as a negative control. See also Movie EV1. Movie EV1 shows the live-cell imaging of FRAP experiments with corresponding plots. The affected chromocenter is highlighted by the dashed red circle on the whole-nucleus view and the zoomed image attached to the whole-nucleus view. Scale bar: 5 and 2 µm in the zoomed image. (**D**) The plot shows the fluorescence recovery after photobleaching curves displayed as mean ± standard error of mean (SEM) for two biological replicates. *n* (MajSat-GFP) = 16, *n* (GFP-H3.1 K36) = 16, and *n* (GFP- H3.1 K36M) = 16. (**E**) Images show the immunofluorescence detection of H3K36me3 and H3K9me3 in the H3.1 GFP-targeted cells from the G1/early S phase fraction. Only the cells with a relatively higher intensity of GFP-H3.1 in the PCH and lower intensity elsewhere in the nucleus were inferred as targeted and selected for analysis. (* marks the PCH). Scale bar: 5 µm. (**F**) The normalized (to DNA) total intensity of H3K9me3 and H3K36me3 was plotted for both conditions using segmented GFP masks (of PCH) in each nuclei. Both pairwise Wilcoxon and Cliff's delta effect size were performed. For H3K9me3, p value = 0.118 (Cliff's Delta = 0.240). For H3K36me3, $p = 0.000011$ (Cliff's Delta = 0.671). Sample sizes (number of nuclei from three biological replicates) are $n = 18$ for H3.1 K36 and $n = 19$ for H3.1 K36M. The boxplot shows the median (line), interquartile range (box: 25th to 75th percentile), and whiskers extending to data within 1.5× the IQR. Statistical significance was performed using the Wilcoxon test (not significant (n.s.) is given for p values ≥0.05; one star (*) for p values <0.05 and ≥0.005; two stars (**) is given for values <0.005 and ≥0.0005, and 0.0005 to 0 are given (***). Source data are available online for this figure.

© The Author(s)

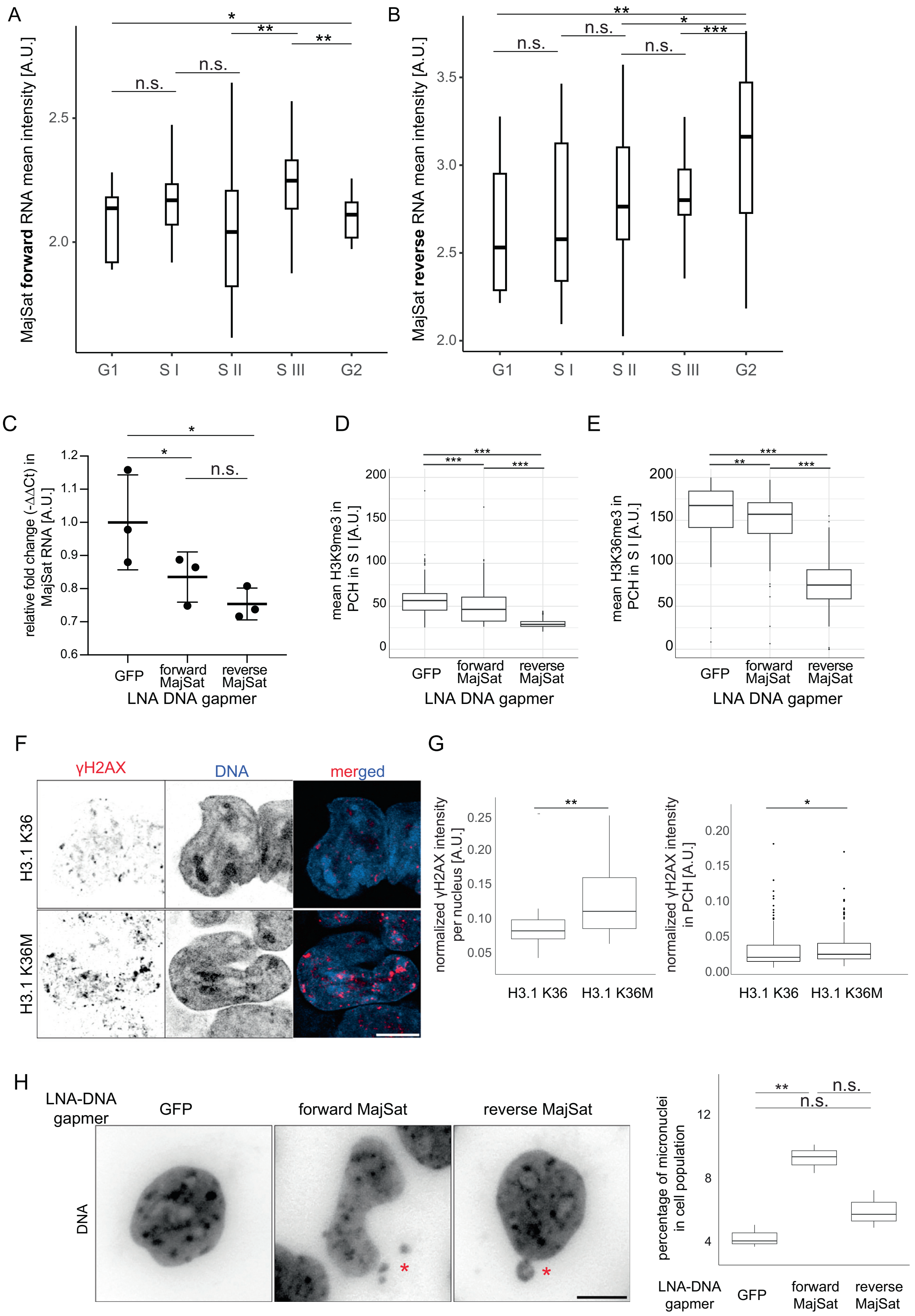
A
MajSat forward RNA mean intensity [A.U.]
n.s.
n.s.
*
**
**
2.5
2.0
G1
S I
S II
S III
G2
B
MajSat reverse RNA mean intensity [A.U.]
**
n.s.
n.s.
n.s.
*
***
3.5
3.0
2.5
2.0
G1
S I
S II
S III
G2
C
relative fold change (-ΔΔCt) in MajSat RNA [A.U.]
1.2
1.1
1.0
0.9
0.8
0.7
0.6
GFP
forward MajSat
reverse MajSat
LNA DNA gapmer
D
mean H3K9me3 in PCH in S I [A.U.]
200
150
100
50
0
GFP
forward MajSat
reverse MajSat
LNA DNA gapmer
E
mean H3K36me3 in PCH in S I [A.U.]
GFP
forward MajSat
reverse MajSat
LNA DNA gapmer
F
γH2AX
DNA
merged
H3.1 K36
H3.1 K36M
G
normalized γH2AX intensity per nucleus [A.U.]
0.25
0.20
0.15
0.10
0.05
H3.1 K36
H3.1 K36M
normalized γH2AX intensity in PCH [A.U.]
0.20
0.15
0.10
0.05
0.00
H3.1 K36
H3.1 K36M
H
LNA-DNA gapmer
GFP
forward MajSat
reverse MajSat
DNA
percentage of micronuclei in cell population
12
8
4
LNA-DNA gapmer
GFP
forward MajSat
reverse MajSat

 © The Author(s)

Figure EV5. Cell cycle-dependent MajSat RNA expression, its epigenetic modulation, and role in maintaining heterochromatin stability.

(**A**) The plot shows the expression of MajSat forward RNA during cell cycle progression inferred from RNA FISH at different cell cycle stages. The statistical test was performed using ANOVA followed by Tukey's honest significant difference (HSD). All p-values are provided in the source data files. The relevant p values are: G1:S I = 0.625911770, S I: S II = 0.040221033, S II: S III = 0.002596268, and S III: G2 = 0.008092236 & G1: G2 = 0.999995863. $n = 170$ from three biological replicates. (**B**) The plot shows the expression of MajSat reverse RNA during cell cycle progression. The statistical test was performed as above, and p values are provided in the source data. The relevant p values are: G1:S I = 0.96182994, S I: S II = 0.95379238, S II: S III = 0.99423587 and S III: G2 = 0.63173138 and G1: G2 = 0.04847357. $n = 176$ from three biological replicates. For both plots in (**A**, **B**), the lower and upper whiskers of the boxplot correspond to the 25th and 75th percentiles, the box to the 50th percentile, and the line depicts the median. (**C**) Plot shows the relative fold change in the respective MajSat RNA upon locked nucleic acid DNA gapmer-mediated interference. Three independent biological replicates were performed, using a pairwise t-test; GFP-Forward MajSat: $p = 0.0130$, GFP-Reverse MajSat: $p = 0.0477$, Forward MajSat-Reverse MajSat = 0.493786605. (**D**) The plot shows the mean H3K9me3 in the PCH upon LNA-DNA-mediated interference. In general, the H3K9me3 level is reduced upon interference of both forward and reverse MajSat RNA and is particularly affected in the reverse. The statistical test was performed using ANOVA followed by Tukey's honest significant difference (HSD). The p values are: GFP-forward = 0.00001, GFP-reverse = 0.0000014, forward-reverse = 0.0000165. (**E**) The plot shows the mean H3K36me3 in the PCH upon LNA-DNA-mediated interference. While in both forward and reverse MajSat interference reduced H3K36me3, the level of H3K36me3 was reduced significantly in the reverse. The statistical test was performed using ANOVA followed by Tukey's honest significant difference (HSD). The p values are: GFP-forward = 0.0002552, GFP-reverse = 0.000001, forward-reverse = 0.00001. For both boxplots (**D**, **E**), the n(GFP) = 14, n(forward) = 14, and n(reverse) = 12 from two biological replicates. (**F**) Images show the γH2AX levels upon targeting of H3.1 with/without K36M. (**G**) The first plot shows the quantification of normalized γH2AX intensity per nucleus, and the second plot shows per PCH area in the H3.1 K36 or H3.1 K36M-targeted cells. The significance test was performed using a pairwise t-test. n (H3.1 K36) = 19, n (KMT) = 21, p value from the pairwise Wilcoxon test p (normalized γH2AX intensity per nucleus) = 0.0025, and p (normalized γH2AX intensity in PCH) = 0.007404. (**H**) Nuclei stained with DAPI in strand-specific interference of the MajSat transcripts. Asterisks (*) mark the micronuclei. The plot shows the percentage of the micronuclei in the cell population upon the interference of MajSat RNA. The significance test was performed using a pairwise t-test; significant difference p value (GFP versus forward MajSat) = 0.0034. n(GFP) = 718, n(forward) = 1225, n(reverse) = 739 from three biological replicates. For plots (**D–H**), the lower and upper whiskers of the boxplot correspond to the 25th and 75th percentiles, the box to the 50th percentile, and the line depicts the median. (not significant (n.s.) is given for p values ≥0.05; one star (*) for p values <0.05 and ≥0.005; two stars (**) is given for values <0.005 and ≥0.0005, and 0.0005 to 0 are given (***); only the significant differences are shown). Scale bar: 5 μm. Source data are available online for this figure.

© The Author(s)
